# Circular RNA circNUP214 Modulates the T Helper 17 Cell Response in Patients With Rheumatoid Arthritis

**DOI:** 10.3389/fimmu.2022.885896

**Published:** 2022-05-24

**Authors:** Huiyong Peng, Jie Xing, Xuehua Wang, Xiangmei Ding, Xinyi Tang, Junli Zou, Shengjun Wang, Yingzhao Liu

**Affiliations:** ^1^ Department of Laboratory Medicine, The Affiliated People’s Hospital of Jiangsu University, Zhenjiang Medical School of Nanjing Medical University, Zhenjiang, China; ^2^ Department of Genetic Toxicology, The Key Laboratory of Modern Toxicology of Ministry of Education, Center for Global Health, School of Public Health, Nanjing Medical University, Nanjing, China; ^3^ Department of Endocrinology, The Fourth Affiliated Hospital of Jiangsu University, Zhenjiang, China; ^4^ Department of Endocrinology, The Affiliated People’s Hospital of Jiangsu University, Zhenjiang Medical School of Nanjing Medical University, Zhenjiang, China; ^5^ Division of Hematology and Internal Medicine, Mayo Clinic, Rochester, MN, United States

**Keywords:** circular RNA, circNUP214, Th17 cells, IL-23R, rheumatoid arthritis

## Abstract

Circular RNAs (circRNAs) are important transcriptional regulators of genome expression that participate in the pathogenesis of human diseases. Mechanistically, circRNAs, as competitive endogenous RNAs (ceRNAs), can sponge microRNAs (miRNAs) with miRNA response elements. A previous study identified that hsa_circ_0089172 (circNUP214) is abnormally expressed in Hashimoto’s thyroiditis. However, the role of circNUP214 in rheumatoid arthritis (RA) remains unclear. In total, 28 RA patients and 28 healthy controls were enrolled in this study. We found that circNUP214 is an abundant and stable circRNA in RA patients that can potentially differentiate RA patients from healthy subjects. Additionally, the elevated levels of IL-23R positively correlated with circNUP214 expression. The knockdown of circNUP214 resulted in the reduction of IL-23R at both transcriptional and translational levels in human CD4^+^ T cells. The proportion of circulating Th17 cells and the transcript levels of IL-17A were increased in RA patients and were both positively correlated with IL-23R expression. Moreover, positive correlations between the transcript levels of circNUP214 and the percentage of Th17 cells and the transcript levels of IL-17A were observed in RA patients. The downregulation of circNUP214 decreased the proportion of Th17 cells and the transcript levels of IL-17A *in vitro*. Furthermore, circNUP214 functioned as a ceRNA for miR-125a-3p in RA patients. Taken together, our results indicate that elevated levels of circNUP214 contribute to the Th17 cell response in RA patients.

## Introduction

Rheumatoid arthritis (RA) is a chronic, inflammatory autoimmune disease characterized by synovial inflammation, bone destruction, and extra-articular symptoms, ultimately resulting in physical disability (1). The disease affects females two to three times more than males and occurs at any age (2). The pathophysiology of RA involves chronic inflammation of the peripheral synovial membrane in multiple joints (3). The relapsing-remitting inflammatory response leads to the progressive destruction of the joint cartilage and juxta articular bone (4). The current advances in the outcomes of RA have been achieved as a consequence of repeated environmental stress over many years, causing dysregulated innate and adaptive immune pathways and eventually immune activation and inflammatory events (5, 6). However, the underlying pathogenesis remains poorly understood. Numerous studies suggest that T helper 17 (Th17) cells play an important role in the pathogenesis of RA (7–9).

The Th17 lineage is a subtype of CD4^+^ T lymphocytes that mainly produces interleukin-17A (IL-17A), an inflammatory cytokine implicated in the pathology of autoimmune inflammatory disorders and the immune response against fungi and some extracellular bacteria (10, 11). In addition to IL-17A, Th17 cells have been shown to coproduce IL-17F, IL-21, IL-22 and IL-26 (12, 13). Retinoic acid-receptor related orphan receptor (RORγt) is an important transcription factor that is sufficient to induce IL-17 production in both human and mouse Th17 cells (14). The differentiation of human Th17 cells depends on the activation of the Janus kinase/signal transducers and activator of transcription 3 (Jak/STAT3) pathway in the presence of IL-6, IL-1β, and IL-23 (15). IL-23 is not involved in the initial differentiation of Th17 cells but is required to expand and stabilize previously differentiated Th17 cells (16). By engaging with its receptor, the interleukin-23 receptor (IL-23R), the cytokine IL-23 induces the differentiation and maturation of Th17 cells (17). The relationship between IL-23 and Th17 cells and the role of IL-23R gene polymorphisms in RA suggests that IL-23R plays an important role in the pathogenesis of RA (18). However, our understanding of Th17 cells in RA remains limited.

Circular RNAs (circRNAs) constitute a novel class of endogenous noncoding RNAs with a covalently closed cyclic structure but without 5’ end caps and 3’ end polyadenylation [poly (A)] tails (19). CircRNAs were first described in RNA viruses in 1976 and subsequently identified in the cytoplasm of eukaryotic cells and yeast mitochondria (20–22). According to the sequences contained in circRNAs, they can be divided into the following three categories: exonic circRNAs, intronic circRNAs and exon-intron circRNAs (23). In contrast to linear RNAs, circRNAs are not easily degraded by ribonuclease R (RNase R) and are more stable *in vivo *(24). Although the majority of these molecules have been identified in the human genome, only a few of their functions have been characterized. Recently, research investigating the roles of circRNAs has focused on immune cells and immune regulation (25–27). CircRNAs are called natural miRNA ‘sponges’ as a single circRNA molecule is able to neutralize several miRNAs and, thus, determine the availability of miRNAs for their posttranscriptional regulation (28). Hsa_circ_0089172 was initially identified in the context of Hashimoto’s thyroiditis (HT), whereas hsa_circ_0089172 expression was increased in HT patients (29), and its corresponding linear RNA was NUP214. However, the underlying role of hsa_circ_0089172 (circNUP214) in the pathogenesis of RA is poorly understood.

In the present study, we aimed to identify the dysregulated expression of circNUP214 in RA patients and investigate whether circNUP214 contributes to the Th17 cell response. Using this approach, we hoped to elucidate the mechanism of Th17 cell proliferation and identify the potential role of circNUP214 in RA.

## Materials and Methods

### Subjects and Samples

Twenty-eight clearly diagnosed adult RA patients (22 females and 6 males), aged 43 to 78 years, were enrolled in this study. All RA patients were diagnosed by their clinical manifestations and an auxiliary examination and met the 2010 revised criteria of the American College of Rheumatology (ACR) and European League Against Rheumatism (EULAR). Among them, 6 patients were negative for both rheumatoid factor (RF) and anti-cyclic citrullinated peptide antibody (anti-CCP-Ab), 12 patients were positive for at least one RF or anti-CCP-Ab, and 10 patients were positive for both RF and anti-CCP-Ab. The acute phase reactants of RA include the erythrocyte sedimentation rate (ESR) and C-reactive protein (CRP). The ESR and CRP were normal in 7 RA patients, while the remaining 21 patients had abnormal CRP or ESR. In addition, one patient was complicated with systemic lupus erythematosus. The disease activity of RA is usually assessed by using the Disease Activity Score 28‐joint count (DAS28), which can be characterized using the ESR (30). The formulas of DAS28-ESR are 0.56 × SQRT (TJC28) + 0.28 × SQRT (SJC28) + 0.70 × ln (ESR) + 0.014 × VAS (31) [TJC28: tender-joint count (0 to 28); SJC28: swollen-joint count (0 to 28); VAS: visual analogue scale (0 to 100 mm)]. The level of disease activity was categorized as follows: a DAS28 less than 2.6 indicated remission; a DAS28 of 2.6 to less than 3.2 indicated low disease activity; a DAS28 of 3.2 to 5.1 indicated moderate disease activity; and a DAS28 greater than 5.1 indicated high disease activity (32). The exclusion criteria were as follows: i) patients with other joint diseases, e.g. osteoarthritis and gout; ii) patients currently participating in clinical trials involving immunotherapy drugs or those who participated in clinical trials involving immunotherapy drugs within 3 months prior to this study; iii) patients with obvious heart, liver, lung or kidney diseases or functional decline; iv) patients with tumours, allergies and haematological diseases; v) patients who have been recently infected; and vi) pregnant or lactating women. Twenty-eight sex- and age- matched healthy adult subjects were included as the controls. All healthy subjects had no history of joint disease, chronic organic diseases, tumours, infectious diseases or other autoimmune diseases. Abnormal serological features, including RF, anti-CCP-Ab, ESR, and CRP, were excluded from the study. The main clinical characteristics of the RA patients and healthy controls are summarized in **Table 1**. Peripheral blood samples were obtained from all patients and healthy controls.

**Table 1:** Clinical features of the patients and the healthy controls included in the study.

Characteristic	RA patients	Healthy controls	Range	P value
Number	28	28	–	–
Gender (M/F)	6/22	8/20	–	0.758
Age (years)	60.89 ± 8.31	59.25±8.18	–	0.459
RF (IU/mL)	120.79±324.70	<9.5	0 to 20	<0.001
CRP (mg/L)	12.19±14.89	1.01±0.31	0 to 5	<0.001
ESR (mm/h)	40.14±26.73	8.50±2.99	0 to 20	<0.001
Anti-CCP-Ab (RU/mL)	468.97±571.02	17.52±1.56	0 to 25	<0.001
DAS28-ESR	4.66±1.74	–	–	–

Data correspond to the arithmetic mean±SD. M, male; F, female. RA, Rheumatoid arthritis; RF, rheumatoid factor; CRP, C-reactive protein; ESR, erythrocyte sedimentation rate; anti-CCP-Ab, anti-cyclic citrullinated peptide antibody; RF of healthy controls were all less than 9.5IU/mL.

All patient sampling procedures were approved by the ethics committee of the Affiliated People’s Hospital of Jiangsu University and collected at the Affiliated People’s Hospital of Jiangsu University after informed consent was obtained from the subjects. All operations in this study adhered to standard biosecurity and institutional safety procedures.

### Laboratory Measurements

The serum concentrations of RF (0 to 20 IU/mL) and CRP (0 to 5 mg/L) were measured by scatter turbidimetry using a BN II instrument (SIEMENS, Marburg, Germany) and an Astep instrument (GOLSITE, Shenzhen, China), respectively. An enzyme linked immunosorbent assay was used to measure the serum levels of anti-CCP-Ab (0 to 25 RU/mL) by using a Benchmark Plus instrument (BIO-RAD, Hercules, USA). The ESR (0 to 20 mm/h) was detected by capillary spectrophotometry using a Roller 20 (Alifax, Padova, Italy).

### Cell Isolation and Purification *In Vitro*

Human peripheral blood mononuclear cells (PBMCs) were isolated by density-gradient centrifugation over Ficoll-Hypaque solution (Haoyang Biological Technology Co., Tianjin, China) and stored at -80°C for quantitative real-time polymerase chain reaction (qRT–PCR). Human CD4^+^ T cells were isolated from PBMCs using human CD4 microbeads (Miltenyi Biotec GmbH, Bergisch Gladbach, Germany) as previously described (33). Human PBMCs and CD4^+^ T cells were cultured with RPMI-1640 medium (Gibco, California, USA) containing 10% foetal bovine serum (Gibco) for transfection. HEK293T cells were maintained in Dulbecco’s modified Eagle’s medium (Gibco) supplemented with 10% foetal bovine serum (Gibco) at 37 °C in 5% CO_2_.

### RNA Isolation, RT–PCR and Real-Time Quantitative RT–PCR 

The total RNA was extracted from the PBMCs with TRIzol reagent (*Invitrogen*, California, USA) according to the manufacturers’ instructions. cDNA was synthesized with oligo-dT and random primers provided in a ReverTraAca^®^qPCR RT kit (Toyobo, Osaka, Japan). Genomic DNA (gDNA) was isolated with a DNA Mini Kit (QIAGEN, Stockach, Germany). After RT–PCR with divergent and convergent primers, agarose gel electrophoresis was performed using the amplified products. qRT–PCR was performed using TaKaRa TB Green Premix Ex Taq II (TaKaRa, Osaka, Japan) to quantify the mRNA and circRNA. The primer sequences are shown in **Supplementary Table 1**. β-actin was used as a reference gene to quantitatively analyse the genes of interest in the study. The data were analysed using Applied Biosystems 7500 Manager software (Thermo Fisher Scientific, Waltham, USA).

### Sanger Sequencing

The total RNA was extracted from human PBMCs and used to synthesize cDNA, which was used in PCR. After PCR, agarose gel electrophoresis was performed using the amplified products. Then, we purified and extracted the DNA from the gel. The DNA samples were sent to Sangon (Sangon Biotech Co., Shanghai, China) for Sanger sequencing of the primer splicing site. The primers for Sanger sequencing: forward 5’- CAGTCAGGCACCAGCTGTAA-3’; reverse 5’- TGGGAGACAGATGACGTTGA-3’.

### Ribonuclease R

The total RNA (2 μg) was isolated from human PBMCs, incubated with 3 U/μg of Ribonuclease R (RNase R) (Epicentre Technologies, Madison, WI, USA) for 10 min at 37°C, and finally inactivated for 10 min at 70°C. After treatment with RNase R, the relative expression levels of circNUP214 and NUP214 mRNA was analyzed by qRT–PCR.

### Nuclear and Cytoplasmic Extraction

Cytoplasmic and nuclear fractions were isolated by a PARIS™ RNA Purification Kit (Thermo Fisher Scientific, Waltham, USA) according to the manufacturers’ instructions. Freshly purified CD4^+^ T cells were lysed in precooled Cell Fractionation Buffer for 10 min. After centrifugation at 500 × g for 5 min at 4°C, the supernatant was collected as the cytoplasmic fraction, and the precipitate was collected as the nuclear fraction. The isolated cytoplasmic and nuclear components were used to detect the circNUP214 levels by qRT–PCR. Lamin B1 and GAPDH were used as nuclear internal control and cytoplasmic internal control, respectively.

### Fluorescence *In Situ* Hybridization

The oligonucleotide-modified probe sequences for circNUP214 and miR-125a-3p were synthesized by GenePharma (GenePharma, Suzhou, China). The fresh CD4^+^ T cell suspension was pipetted onto autoclaved glass slides. The probes and SA-CY3 were proportioned to prepare the probe mixture. Then, hybridization was performed at 37°C overnight in a dark environment. After washing thrice with 20× SCC/–hybridization buffer for 5 min, the slides were incubated with DAPI for 20 min. Images were acquired using fluorescence microscopy (OLYMPUS FV1000 confocal microscopy, Japan). The probe sequences were as follows: circNUP214: 5’ – TGATCGAGACAGGCTGGCCCATGGCTGTAGAAGGGGT - 3’, miR-125a-3p: 5’ - GGCTCCCAAGAACCTCACCTGT - 3’.

### Flow Cytometry Analysis

Fresh PBMCs or CD4^+^ T cells were resuspended in RPMI-1640 medium supplemented with 10% foetal bovine serum and stimulated with 50 ng/ml phorbol myristate acetate (PMA; Sigma–Aldrich) and 1 µg/ml ionomycin (Sigma–Aldrich) for 2 hours. Subsequently, the cells were incubated for an additional 4 hours in the presence of 1 µg/ml brefeldin-A (eBioscience, San Diego, USA) at 37°C with 5% CO_2_. The suspended cells were subsequently stained with relevant mAbs, including phycoerythrin-cyanin 5 (PE-Cy5)-conjugated anti-human CD3 mAb, fluorescein isothiocyanate (FITC)-conjugated anti-human CD8 mAb (eBioscience), PE-conjugated anti-human IL-17A mAb, and PE-conjugated anti-human IL-23R mAb (R&D Systems, Minnesota, USA). FlowJo 10 (Stanford University, San Francisco, USA) was used to analyse the data. In this study, we defined CD3^+^ CD8^-^ IL-17A^+^ cells as Th17 cells.

### RNA Interference and Transfection

Small interfering RNA (siRNA) (RiboBio, Guangzhou, China) was designed to chemically modify target-specific circNUP214 in cells. Nonspecific scramble siRNA was used as a negative control (NC) (RiboBio). Fresh human CD4^+^ T cells were transfected with circNUP214 siRNA or NC at 100 nM using Entranster-R (Engreen Biosystem, Co., Ltd., Beijing, China) according to the manufacturers’ instructions in the presence of 0.5 μg/ml functional anti-human CD3 mAb plus 2 μg/ml functional anti-human CD28 mAb (Miltenyi Biotec GmbH) before restimulation (34). The cells were harvested for flow cytometry analysis and qPT–PCR.

### Luciferase Reporter Assay

The wild-type (WT) sequence of circNUP214 containing the miR-125a-3p binding sites was generated by Sangon (Sangon Biotech Co.). Mutations were performed in the binding sites of miR-125a-3p. The WT and mutated sequences of the circNUP214 were cloned into the vector psiCHECK-2 (Promega, Madison, USA). Then, a dual-luciferase reporter plasmid and miR-125a-3p mimics or miR-NC were cotransfected into HEK293T cells using Lipofectamine 3000 reagent (Thermo Fisher Scientific, Waltham, MA, USA) and Entranster-R in 24-well plates. Forty-eight hours after the transfection, the relative luciferase activity was measured as the ratio between Firefly and Renilla luciferase activities using a Dual-Luciferase Reporter Assay System (Promega, Madison, USA) according to the manufacturers’ instructions.

### Statistical Analysis

GraphPad Prism version 5 software (GraphPad Software, Inc., San Diego, USA) was used to process and analyse the data. A student’s unpaired t-test was used for the comparisons of two groups when the variables passed the normal distribution test. A Mann-Whitney U test was performed to analyse the differences between two groups with nonnormally distributed data. A one-way ANOVA test was used for the comparisons of multiple groups. Tukey’s test was applied for pair-to-pair comparisons of multiple groups after the ANOVA. The correlations between the variables were determined by the Pearson correlation coefficient when the variables passed the normal distribution test. The correlations between the nonnormally distributed variables were calculated by Spearman correlation coefficient. Receiver operating characteristic (ROC) curves and the area under ROC curve (AUC) were calculated to evaluate the potential diagnostic value of circNUP214. Sensitivity was used as the Y-axis to represent the true positive rate, while 100%−specificity% was used as the X-axis to represent the false-positive rate. *p* < 0.05 was considered indicative of a statistically significant difference. (*p < 0.05, **p < 0.01, ***p < 0.001).

## Results

### Characterization of circNUP214

**Figure 1:**
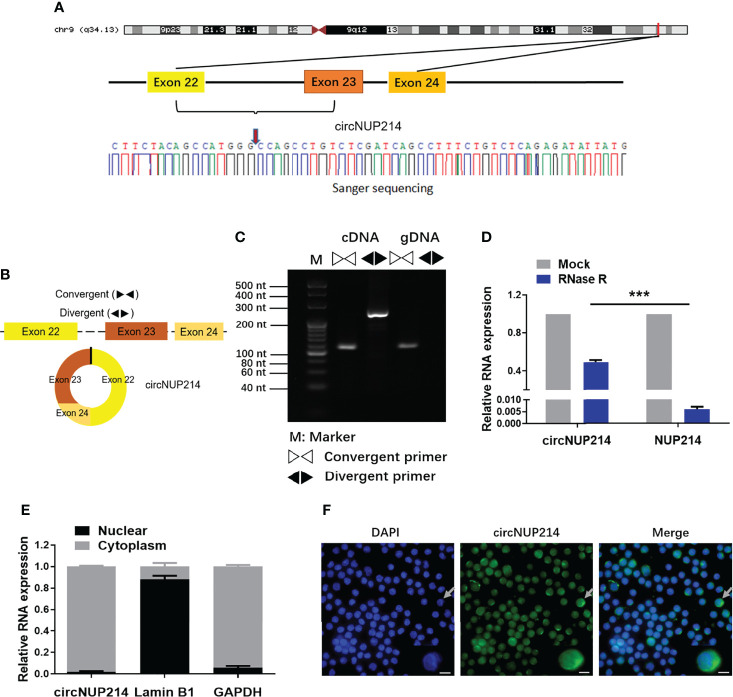
Characterization of circNUP214. **(A)** Genomic position of circNUP214 on the human chromosome and Sanger sequencing following PCR confirmed the ‘head-to-tail’ splicing of circNUP214 in PBMCs. The red arrow represents backsplicing junction site. **(B)** Convergent and divergent primers of circNUP214 were designed to amplify the linear or back-splicing products. **(C)** qRT–PCR products with divergent primers showing the circularization of circNUP214. cDNA: complementary DNA. gDNA: genomic DNA. **(D)** The relative expression of circNUP214 and NUP214 mRNA after the treatment with RNase R in PBMCs was determined by qRT–PCR. **(E)** Cytoplasmic and nuclear fractionation experiment showed that circNUP214 was mainly localized in the cytoplasm. GAPDH and Lamin B1 were applied as positive controls in the cytoplasm and nucleus, respectively. Data represent mean ± S.D. from three independent experiments. **(F)** RNA fluorescence *in situ* hybridization of circNUP214. Nuclei were stained with DAPI. Scale bar, 25 µm. ***p < 0.001.

The circNUP214 transcript (chr9: 134049441-134053797) is an exonic circular RNA located on chromosome 9q34. First, we characterized circNUP214 by Sanger sequencing, and the results confirmed that circNUP214 was head-to-tail spliced (**Figure 1A**). After the RT–PCR analysis, the divergent primers were capable of generating the circular isoform of circNUP214 from cDNA rather than from gDNA in PBMCs (**Figures 1B, C**). Then, we compared the resistance of circNUP214 to the corresponding linear RNA NUP214 to RNase R treatment. The resistance to digestion by the RNase R treatment confirmed that this RNA species harbours a circular RNA structure (**Figure 1D**). The nuclear and cytoplasmic fractionation experiments showed that circNUP214 was mainly present in the cytoplasm (**Figure 1E**). The FISH examination revealed that circNUP214 was mainly localized in the cytoplasm (**Figure 1F**). These results revealed that circNUP214 was a stable circRNA expressed in the cytoplasm.

### Increased circNUP214 Expression in RA Patients

**Figure 2:**
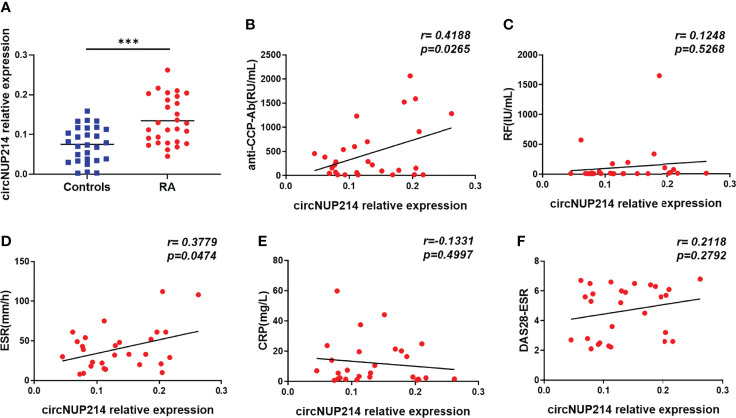
Increased circNUP214 expression in RA patients. **(A)** The transcript levels of circNUP214 in PBMCs from 28 RA patientsand 28 controlswere determined by qRT–PCR. The correlations between circNUP214 expression and the concentrations of anti-CCP Ab **(B)**, RF **(C)**, ESR **(D)**, CRP **(E)**, and DAS28-ESR score **(F)** in 28 RA patients are shown. Each data point represents an individual subject, and the horizontal lines show the mean. ***p < 0.001.

To investigate circNUP214 expression in RA patients, peripheral blood was obtained from RA patients and healthy subjects. As shown in **Figure 2A**, the transcript levels of circNUP214 were upregulated in PBMCs from RA patients compared with those from healthy controls. RF, anti-CCP-Ab, ESR, and CRP are the clinical diagnostic hallmarks of RA. We examined the relationship between circNUP214 and these parameters. The levels of circNUP214 positively correlated with the serum levels of anti-CCP-Ab (r = 0.4188; *p* = 0.0265) (**Figure 2B**) and the levels of ESR (r = 0.3779; *p* = 0.0474) (**Figure 2D**) but not with the levels of RF (r = 0.1248; *p* = 0.5268) (**Figure 2C**) and CRP (r = -0.1331; *p* = 0.4997) (**Figure 2E**). In addition, we analysed the relationship between circNUP214 expression and the activity index of RA and found that there was no correlation between circNUP214 expression and the DAS28-ESR score (r = 0.2118; *p* = 0.2792) (**Figure 2F**). These data demonstrated that abnormal circNUP214 was associated with the process of RA.

### Effect of circNUP214 on IL-23R Expression in RA Patients

**Figure 3:**
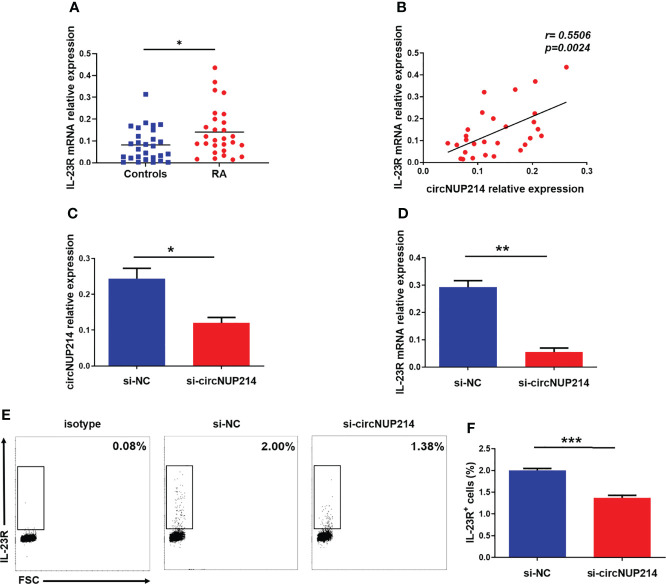
Effect of circNUP214 on IL-23R expression in RA patients. **(A)** The transcript levels of IL-23R mRNA in PBMCs from 28 RA patients and 28 controls were determined by qRT–PCR. **(B)** The correlation between circNUP214 expression and IL-23R levels in 28 RA patients. Each data point represents an individual subject, and the horizontal lines show the mean. **(C)** The transcript levels of circNUP214 were detected by qRT–PCR after transfection with circNUP214-specific siRNA and negative control (si-NC) in human CD4^+^ T cells. **(D)** IL-23R mRNA expression was detected by qRT–PCR after transfection with si-circNUP214 and si-NC. **(E, F)** The percentage of IL-23R^+^ cells were analysed by flow cytometry. Data represent mean ± S.D. from three independent experiments. *p < 0.05; **p < 0.01; ***p < 0.001.

To analyse the role of circNUP214 in IL-23R expression in RA patients, the transcript levels of IL-23R were first determined by qRT–PCR. Elevated levels of IL-23R were found in PBMCs from RA patients (**Figure 3A**). Moreover, a significantly positive correlation was observed between the relative expression of circNUP214 and the transcript levels of IL-23R (r = 0.5506; *p* = 0.0024) (**Figure 3B**). Then, si-circNUP214 and si-NC were transfected into human CD4^+^ T cells. The downregulated expression of circNUP214 with siRNA (**Figure 3C**) resulted in a reduction in the transcript levels of IL-23R (**Figure 3D**), which consequently caused the reduction of the proportion of IL-23R^+^ cells compared with that in the si-NC group (**Figures 3E, F**). Altogether, these results indicate that circNUP214 regulated IL-23R expression in RA patients.

### Correlation Between IL-23R Expression and Circulating Th17 Cells in RA Patients

**Figure 4:**
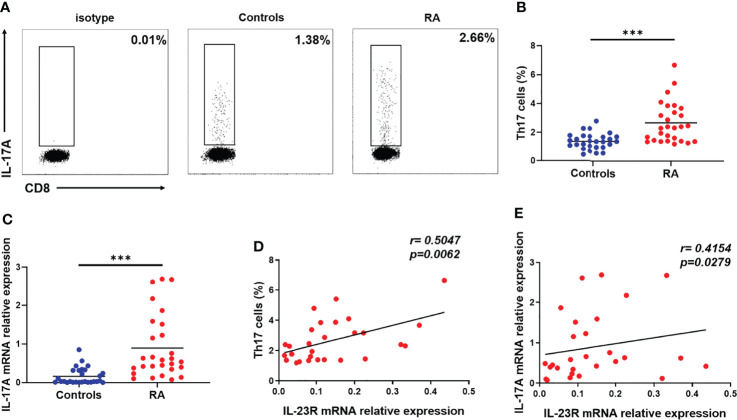
Correlation between IL-23R expression and circulating Th17 cells in RA patients. Fresh peripheral blood was collected, PBMCs were subsequently isolated from peripheral blood within four hours, and the proportion of Th17 cells was analysed by flow cytometry. **(A)** Representative flow cytometry dot plots of Th17 cells in RA patients and controls. Values in the upper left rectangular region corresponded to the proportion of Th17 cells. CD3^+^ CD8^-^ IL-17A^+^ cells were gated as Th17 cells. **(B)** The difference in the proportion of Th17 cells between 28 RA patients and 28 controls is shown. **(C)** The relative expression of IL-17A mRNA in PBMCs from 28 RA patients and 28 controls was determined by qRT–PCR. The correlation between IL-23R expression and the percentage of Th17 cells **(D)** and IL-17A levels **(E)** in 28 RA patients. Each data point represents an individual subject, horizontal lines show the mean. ***p < 0.001.

IL-23R is an important molecule for the development of Th17 cells. To assess the relationship between IL-23R and Th17 cells in RA patients, we gated on CD3^+^ CD8^-^ IL-17^+^ lymphocytes to distinguish Th17 cells from PBMCs due to the degradation of membrane CD4 on human PBMCs in response to PMA (35) (**Figure 4A**). Compared to the controls, the proportion of Th17 cells was significantly increased in patients with RA (**Figure 4B**). The disruption of IL-17A expression in RA patients was sequentially analysed by qRT–PCR. As shown in **Figure 4C**, IL-17A expression in RA patients was higher than that in controls. Moreover, positive correlations between the transcript levels of IL-23R and the percentage of Th17 cells (r = 0.5047; *p* = 0.0062) (**Figure 4D**) and the transcript levels of IL-17A r 0.4154; *p* = 0.0279) (**Figure 4E**) were observed in RA patients. These results indicate that IL-23R plays a crucial role in increasing circulating Th17 cells in RA patients.

### Influence of circNUP214 on Th17 Cells in RA Patients

**Figure 5:**
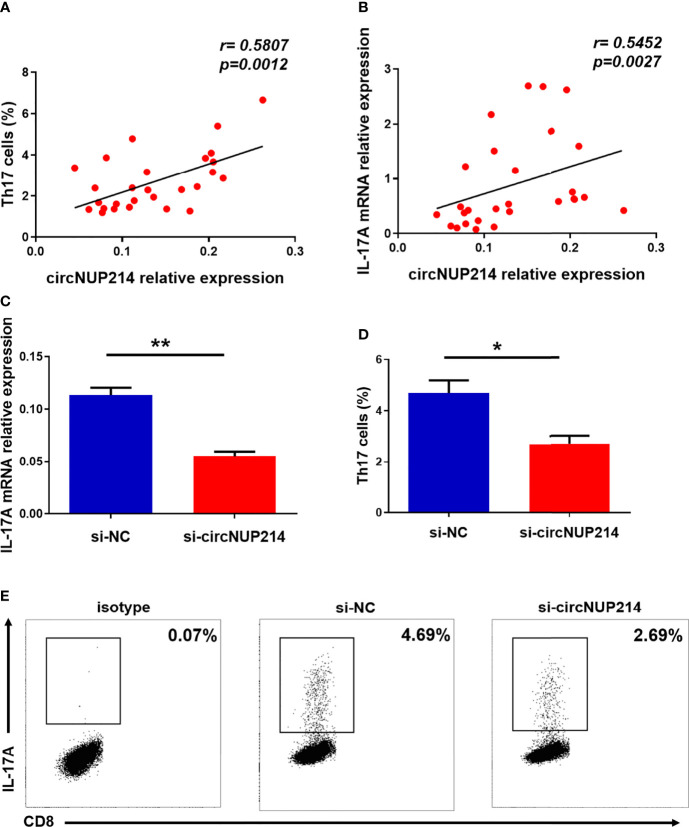
Influence of circNUP214 on Th17 cells in RA patients. **(A)** The correlation between the transcript levels of circNUP214 and the proportion of Th17 cells in 28 RA patients. **(B)** The correlation between the transcript levels of circNUP214 and the transcript levels of IL-17A in 28 RA patients. Each data point represents an individual subject. Human CD4^+^ T cells were purified from PBMCs by magnetic beads and transfected with circNUP214-specific siRNA and NC (100 nM) in the presence of functional anti-human CD3 mAb and anti-human CD28 mAb for 24 h to detect the transcript levels of IL-17A and 48 h before restimulation to determine the proportion of Th17 cells. **(C)** The relative expression of IL-17A was detected after transfection with circNUP214 siRNA and si-NC. **(D, E)** The percentage of Th17 cells was analysed by flow cytometry. Data represent mean ± S.D. from three independent experiments. *p < 0.05; **p < 0.01.

To address the possibility that circNUP214 contributes to increased Th17 cells in RA patients, we assessed the relationship between circNUP214 and Th17 cells. Positive correlations were observed between the circNUP214 levels and the percentage of Th17 cells (r = 0.5807; *p*= 0.0012) (**Figure 5A**) and the transcript levels of IL-17A (r = 0.5452; *p* = 0.0027) (**Figure 5B**). To further investigate the potential role of circNUP214 in Th17 cells, circNUP214 siRNA and NC were transfected into human purified CD4^+^ T cells. We found that the knockdown of circNUP214 dramatically reduced IL-17A expression (**Figure 5C**). Consistently, the percentage of Th17 cells was also downregulated upon the circNUP214 depletion (**Figures 5D, E**). Collectively, these data indicate that circNUP214 contributes to the Th17 cell response in RA patients.

### CircNUP214 as a miR-125a-3p Sponge in RA Patients

**Figure 6:**
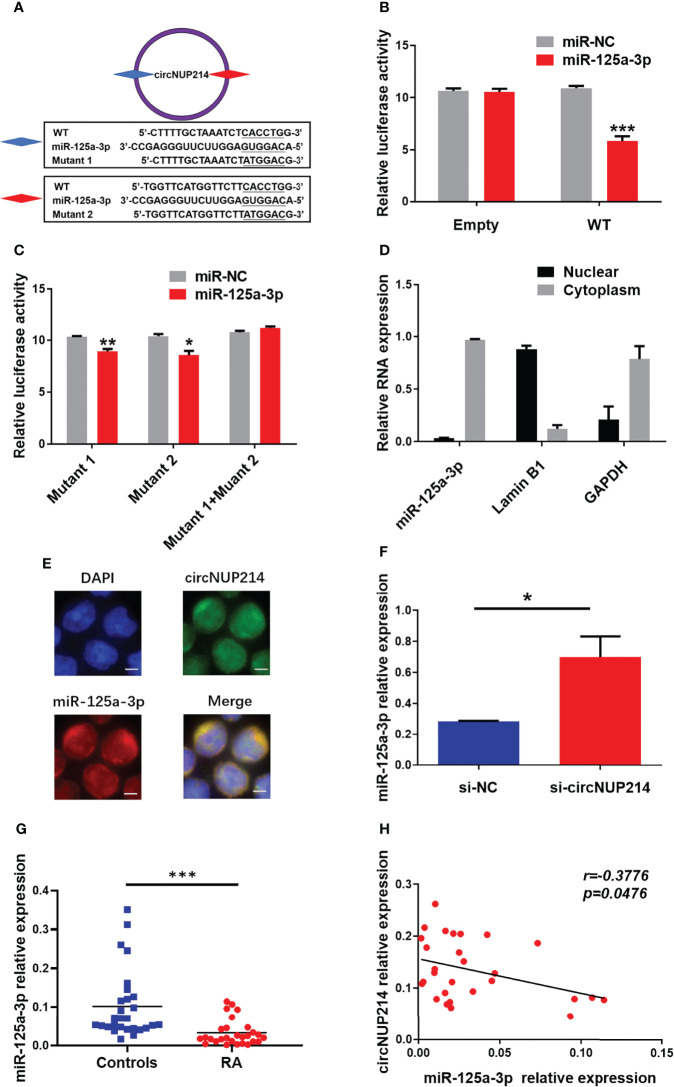
CircNUP214 as a miR-125a-3p sponge in RA patients. **(A)** The binding sites of miR-125a-3p on the sequence of circNUP214 and the mutated sequences of circNUP214. **(B)** Luciferase activity of a reporter carrying WT and empty groups cotransfected into HEK293T cells with miR-125a-3p mimics and miR-NC. **(C)** Luciferase screening assay showed the luciferase activity of mutants 1, 2 and 1 + 2 transfected with miR-125a-3p mimics and miR-NC. Mutant 1 + Mutant 2 represents a mutant with both mutated sites. **(D)** The distribution of miR-125a-3p was determined by cytoplasmic and nuclear fractionation experiments. GAPDH and Lamin B1 were applied as positive controls in the cytoplasm and nucleus, respectively. **(E)** The colocalization of circNUP214 and miR-125a-3p in CD4^+^ T cells was shown by FISH. Scale bar, 25 µm. **(F)** The levels of miR-125a-3p in human CD4^+^ T cells transfected with si-circNUP214 and si-NC. Data represent mean ± S.D. from three independent experiments. **(G)** The relative expression of miR-125a-3p in PBMCs from 28 RA patients and 28 controls were determined by qRT–PCR. **(H)** The correlation between the levels of circNUP214 and the levels of miR-125a-3p in 28 RA patients. Each data point represents an individual subject, and the horizontal lines show the mean. *p < 0.05; **p < 0.01; ***p < 0.001.

One of the main roles of circRNAs in the cytoplasm is to bind miRNAs as competing endogenous RNAs (ceRNAs). The circNUP214 transcript has two binding sites for miR-125a-3p (**Figure 6A**). To determine whether miR-125a-3p binds circNUP214, we constructed luciferase reporter minigenes containing wild-type (WT) circNUP214 or mutant circNUP214 (Mutant 1 and Mutant 2). Luciferase reporters were transfected into human 293T cells with miR-125a-3p mimics or miR-NC. The transfection with miR-125a-3p was found to reduce the luciferase activity of WT by approximately 50% compared with the miR-NC transfection (**Figure 6B**). In addition, miR-125a-3p dramatically inhibited the luciferase activity of Mutant 1 or Mutant 2, but not that of the reporter with a mutated reporter unable to bind miR-125a-3p (Mutant 1 + Mutant 2) (**Figure 6C**). The nuclear and cytoplasmic fractionation showed that miR-125a-3p was mainly localized in the cytoplasm (**Figure 6D**). The interaction between circNUP214 and miR-125a-3p was further demonstrated by FISH assays, and the results showed that circNUP214 and miR-125a-3p were colocalized in the cytoplasm (**Figure 6E**). We further found that the knockdown of circNUP214 significantly increased miR-125a-3p expression in CD4^+^ T cells (**Figure 6F**). Then, we detected miR-125a-3p expression in PBMCs from RA patients and found that miR-125a-3p expression was downregulated in RA patients compared with that in controls (**Figure 6G**). Furthermore, a significantly inverse correlation between miR-125a-3p and circNUP214 was found in RA patients (r = -0.3776; *p* = 0.0476) (**Figure 6H**). These results suggest that circNUP214 exerts its function in RA by sponging miR-125a-3p.

### Potential Value of circNUP214 in RA

To evaluate the potential value of circNUP214 in RA patients, a ROC curve analysis was performed to analyse the levels of circNUP214. As shown in **Supplementary Figure 1**, the ROC curve revealed that circNUP214 was able to distinguish the RA patients from the healthy controls. The AUC was up to 0.76 [95% confidence interval (CI) = 0.64-0.88, *p* < 0.001], and the sensitivity, specificity, Youden index, and likelihood were 42.86%, 96.43%, 0.39, and 12, respectively.

### Discussion

In this study, we provide the first line of evidence suggesting that circNUP214 is an abundant and stable circRNA that promotes the Th17 cell response by regulating IL-23R in RA patients. The ROC curve analysis of circNUP214 and the correlation between circNUP214 and disease severity indicate that circNUP214 plays a pivotal role in RA. Intriguingly, circNUP214 may play a critical role by sponging miR-125a-3p in the pathogenesis of RA.

CircRNAs are covalent closed-loop RNA molecules discovered more than four decades ago (36). However, by approximately 2013, numerous circRNAs and their biological functions were identified as a consequence of the rapid development of bioinformatics and high-throughput sequencing technologies (37, 38). CiRS-7, also named cerebellar degeneration-related protein 1 transcript antisense (CDR1as), was first identified in human and mouse neuronal tissues (39). This circRNA contains more than 70 selectively conserved miR-7 target sites and functions by binding miR-7 as a negative regulator, ultimately leading to increased levels of miR-7 targets (39, 40). Subsequently, increasing evidence indicates that various circRNAs are involved in many physiological and pathological processes (41, 42). Current studies investigating circRNAs highlight their role in human diseases, including neurological diseases, cancer, immune disorders, and cardiovascular diseases (43, 44). RA is an autoimmune disorder with aberrant regulation and function of immune cells (45). In recent years, abnormal circRNAs have been extensively studied as potential biomarkers of RA (46, 47). A little-noticed circRNA, called circNUP214, located at chr9: 134049441-134053797, and its linear gene is NUP214. The genomic length of circNUP214 is 4356 bp. We found that circNUP214 expression was significantly increased in peripheral blood from RA patients and was associated with laboratory parameters. To further investigate the potential value of circNUP214 in RA, an ROC curve analysis was conducted, and the sensitivity and specificity indices indicated that circNUP214 could be used as a potential auxiliary indicator of immune disorder in RA, but larger cohorts of RA patients should be enrolled in future studies.

The function of circNUP214 in RA remains enigmatic. Th17 cells, which interact with stromal and immune cells or induce and activate osteoclasts in RA, contribute to the synovial joint inflammatory response and irreversible joint and cartilage destruction (48). The proportion of Th17 cells and IL-17A expression were increased in PMBCs from patients with RA. IL-23R is an important molecule involved in the development of Th17 cells (49). Our data also revealed that the elevated levels of IL-23R were positively correlated with the percentage of Th17 cells and the transcript levels of IL-17A in RA patients. Our previous study suggested that circNUP214 regulated the transcription levels of IL-23R in PBMCs (29). Based on this finding, we hypothesized that circNUP214 might contribute to Th17 cells by regulating IL-23R in RA patients. As expected, positive correlations were found between the circNUP214 levels and the proportion of Th17 cells and IL-17A levels in RA patients. The knockdown of circNUP214 significantly inhibited IL-17A expression, which consequently caused a reduction in Th17 cells *in vitro*. To further elucidate the role of circNUP214 in Th17 cells in RA patients, the relationship between circNUP214 and IL-23R was analysed. Our data indicate that the circNUP214 levels positively correlated with IL-23R expression in RA patients. Meanwhile, the knockdown of circNUP214 decreased the transcript levels of IL-23R and the numbers of IL-23R^+^ cells in human CD4^+^ T cells. Collectively, these data suggested that circNUP214 is involved in the development of Th17 cells by regulating IL-23R expression in RA patients. However, the underlying mechanism by which circNUP214 promotes IL-23R expression remains unknown.

Till date, the well-studied functions of circRNAs have been identified. First, nuclear retained circular RNAs regulate gene expression by participating in alternative splicing and transcription (50). Second, circRNAs exert their effects by binding proteins (50). Third, circRNAs can act as “molecular sponges” to competitively bind miRNAs (39). Finally, some circRNAs can be translated into encoded proteins (51). CircNUP214 was mainly expressed in the cytoplasm, suggesting the possibility that it could act as a sponge for miRNAs. The sequence of circNUP214 contained the binding sites of miR-125a-3p. The nuclear and cytoplasmic fractionation studies and FISH examination showed that miR-125a-3p was mainly enriched in the cytoplasm. Based on these findings, we hypothesized that miR-125a-3p might bind the sequence of circNUP214, which was subsequently confirmed by luciferase activity assays. The downregulation of circNUP214 increased miR-125a-3p expression *in vitro*. We also found that miR-125a-3p expression was significantly decreased in PBMCs from RA patients. An inverse correlation between the levels of miR-125a-3p and the levels of circNUP214 was observed in RA patients. Our previous study demonstrated that miR-125a-3p directly targets IL-23R (52). Here, we showed that circNUP214, as the sponge of miR-125a-3p, promotes IL-23R expression, suggesting that miR-125a-3p may be an intermediate point and that circNUP214 functions in Th17 cells may act as a ceRNA for miR-125a-3p. However, much work remains to be performed.

As generally accepted, RF and anti-CCP antibodies are important markers for the diagnosis and prognosis of RA (53). Numerous clinical data have shown that RF is not specific for RA diagnosis, and approximately 30% to 45% of patients with RA do not have RF (54). Anti-CCP antibody is an index of the prognosis of RA and is extremely specific for RA diagnosis (55). In this study, our data showed a positive correlation between the transcript levels of circNUP214 and the serum levels of anti-CCP antibody but not with the serum levels of RF, which has been indirectly proven by the positive correlation between the proportion of Th17 cells and the levels of anti-CCP antibody but not RF (56). However, the reasons for this difference also deserve further exploration. The acute phase reactants of RA include ESR and CRP according to ACR/EULAR 2010. Intriguingly, the correlation between CRP or ESR and circNUP214 was inconsistent. One possible explanation for this phenomenon is that there are many factors influencing their measurement. A large observational study noted discordant ESR and CRP values in 26% of RA patients (57). Another possible explanation for this phenomenon is that the sample size included in the study is insufficient. In addition, there was no correlation between circNUP214 expression and the activity index of RA. We think that circNUP214 may be a potential auxiliary indicator of immune disorder. However, the specific mechanisms remain to be further investigated.

There are limitations in this study. One limitation of our study is the approach to setting CD3^+^ CD8^−^ cells as CD4^+^ T cells after stimulation with PMA and ionomycin in PBMCs. This reverse gate method cannot exclude the influence of γδ T17 cells (IL-17A–producing γδ T cells) (58), which may result in a higher count of Th17 cells than actual Th17 cells. This may further affect the correlation between circNUP214 and Th17 cells in RA patients. Further study is needed to define Th17 cells with more comprehensive markers or better stimulators. Another limitation is that the number of volunteers was small. Further study is needed to investigate circNUP214 expression and its potential value in larger cohorts of RA patients. Furthermore, our experiments have thus far been conducted only in humans. Further animal model experiments should be conducted to increase our comprehension of the detailed mechanisms and specific functions of circNUP214 in an RA model. In summary, our results demonstrate that circNUP214 is an elevated circRNA that contributes to the pathogenic role of the Th17 cell response in RA patients.

## Data Availability Statement

The original contributions presented in the study are included in the article/supplementary files, further inquiries can be directed to the corresponding author/s.

## Ethics Statement

The studies involving human participants were reviewed and approved by The Ethics Committee of the Affiliated People’s Hospital of Jiangsu University (K-20190057-Y). The patients/participants provided their written informed consent to participate in this study. Written informed consent was obtained from the individual(s) for the publication of any potentially identifiable images or data included in this article.

## Author Contributions

HP, JX, and XW, carried out the experiments, analysed the data, and wrote the manuscript. XD and JZ helped with the experiments and analysed the data. XT participated in the design of the experiments. YL and SW planned the experiments and supervised all work in this paper. All authors discussed the results and commented on the manuscript. All authors contributed to the article and approved the submitted version.

## Funding

This work was supported by National Natural Science Foundation of China (Grant No. 81800698), Zhenjiang Sixth Phase 169 Project Training Fund Support Project (No. 28 of academic hard-core personnel research project), and Zhenjiang Science and Technology Planning Project (Grant No.SH2021026, SH2021059).

## Conflict of Interest

The authors declare that the research was conducted in the absence of any commercial or financial relationships that could be construed as a potential conflict of interest.

## Publisher’s Note

All claims expressed in this article are solely those of the authors and do not necessarily represent those of their affiliated organizations, or those of the publisher, the editors and the reviewers. Any product that may be evaluated in this article, or claim that may be made by its manufacturer, is not guaranteed or endorsed by the publisher.

## Supplementary Material

Supplementary Figure 1 The potential value of circNUP214 in RA. ROC curve analysis of circNUP214 was performed to distinguish the RA patients from the healthy volunteers. The area under the ROC curve (AUC) was up to 0.76.
