# Supplementary figures and images for: Circular RNA circNUP214 Modulates the T Helper 17 Cell Response in Patients With Rheumatoid Arthritis
Source: Front Immunol. 2022 May 24;13:885896. doi: 10.3389/fimmu.2022.885896 (PMC9170918; doi:10.3389/fimmu.2022.885896)

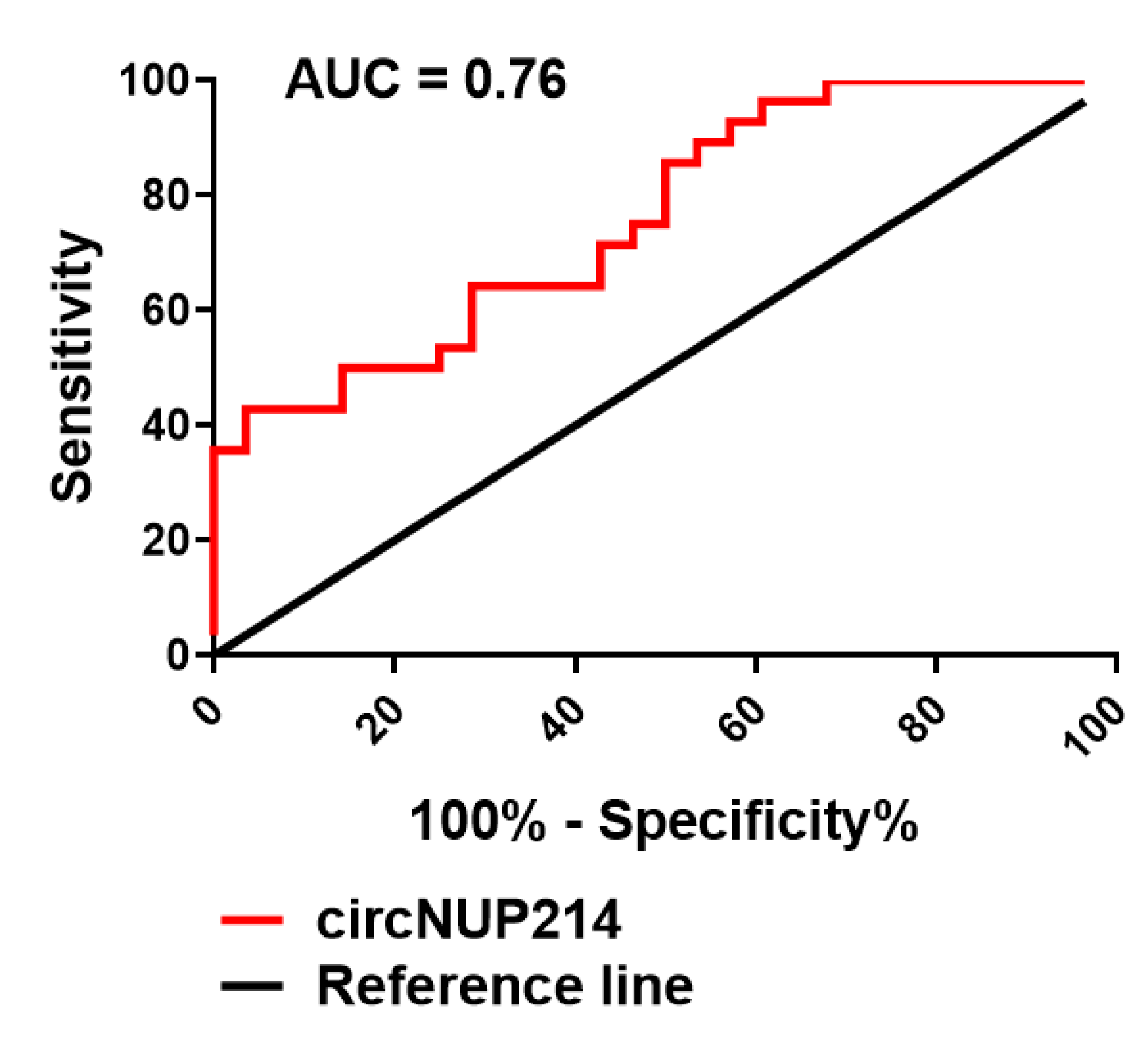

Supplement: Supplementary Figure 1 — The potential value of circNUP214 in RA. ROC curve analysis of circNUP214 was performed to distinguish the RA patients from the healthy volunteers. The area under the ROC curve (AUC) was up to 0.76. [file Image_1.tiff]
