# Supplementary material for: Circular RNA circNUP214 Modulates the T Helper 17 Cell Response in Patients With Rheumatoid Arthritis
Source: Front Immunol. 2022 May 24;13:885896. doi: 10.3389/fimmu.2022.885896 (PMC9170918; doi:10.3389/fimmu.2022.885896)
Supplement: Supplementary file 2 [file Table_1.docx]

**Table S1. Primer sequences of the genes in the study**

| **PRIMER ID** | **NUCLEOTIDE** | **SEQUENCE (5'-3')** | |
| --- | --- | --- | --- |
| CircNUP214 | NM_005085 | Forward | CCGATAGCACAATGCTTGCC |
|  |  | Reverse | ATGGCTGTAGAAGGGGTTGC |
| IL-17A | NM_002190.3 | Forward | CAAGACTGAACACCGACTAAG |
|  |  | Reverse | TCTCCAAAGGAAGCCTGA |
| IL-23R | NM_144701.3 | Forward | TCAAGAAACAGGCAAAAGGTAC |
|  |  | Reverse | GACAACATAACAGCAAAGACGAT |
| β-actin | NM_001101.5 | Forward | GAGTGTGGAGACCATCAAGGA |
|  |  | Reverse | TGTATTGCTTTGCGTTGGAC |
| miR-125a-3p | MIMAT0004602 | Forward | GGCGACAGGTGAGGTTCTT |
|  |  | Reverse | GCAGGGTCCGAGGTATTC |
| Lamin B1 | NM_001198557.2 | Forward | GGAAGTGAGCGGCATCAAGGAG |
|  |  | Reverse | ACTGACCTGGCACGGAGATCC |
| GAPDH | NM_00189745.3 | Forward | ACAACTTTGGTATCGTGGAAGG |
|  |  | Reverse | GCCATCACGCCACAGTTTC |
| NUP214 | NM_001318324.2 | Forward | TGAGAAGCCAGGTGACAGTG |
|  |  | Reverse | GTCAGAAGTTTGCGGAGGAG |
